# *Botsurisōronsō* – The ‘Submerged Ideals-Debate’ in Meiji Japan by Tsubouchi Shōyō and Mori Ōgai

**DOI:** 10.1515/cat-2024-0011

**Published:** 2025-04-17

**Authors:** Naomi Charlotte Fukuzawa

**Affiliations:** International Office, 38928 University of Applied Sciences Potsdam, Potsdam, Germany

**Keywords:** modern Japanese literature, literary theory, romanticism and realism

## Abstract

In the early 1890s, the *Botsurisōronsō* debate on ‘submerged ideals’ took place between the literary theorist, translator and academic Tsubouchi Shōyō (1859–1935) and the writer, translator and medical researcher Mori Ōgai (1862–1922). The discussion was carried out by Shōyō in the pages of the literary journal *Waseda bungaku*, which he had founded as professor of Waseda University, and Ōgai in the journal *Shigarami Zōshi,* which Ōgai had founded. Initially responding to Shōyō’s advocacy of Shakespeare, Ōgai addressed the question of whether modern Japanese literature should absorb and reflect metaphysical ideals or not. Recently returned from a trip to Prussia, Ōgai introduced the aesthetic philosophy of Eduard von Hartmann, claiming it as compatible with Shōyō’s subdivision of the novel into three schools. The opposition of the two eminent literary figures was also reflected in Shōyō’s English-style philosophy and Ōgai’s German metaphysical idealism in the Romantic tradition. This means that Shōyō emphasized a poetics of literature emulating photographic portrayal as well as the incorporation of the Buddhist aesthetics of *mono no aware*, while Ōgai advocated an idealistic understanding of ideas in the German Romantic tradition. This debate showed the Japanese public for the first time the role of modern literature, *bungaku*, as notions of literary fiction were arising in Meiji.

## 1 The ‘Submerged Ideals’ Debate (*Botsurisōronsō*) by Tsubouchi Shōyō and Mori Ōgai

In 1891 and 1892, a debate called *Botsurisōronsō* (没理想論争) on ‘submerged ideals’ was carried out between Tsubouchi Shōyō 坪内逍遥 (1859–1935) and Mori Ōgai 森鴎外 (1862–1922) around the question whether modern literature should reflect certain ideals or not.1The named sources by Shōyō and Ōgai, mostly in Waseda bungaku (WB) and Shigarami zōshi (SZ), are not published as English translations. For this article, I cite my own English translations in reference to the published Japanese originals. The debate emerged from a critical discussion of Shakespeare, which lead to a controversy about whether modern Japanese literature should adhere to the English school of Realism (*shajitsuh*a 写実派) or German-style Romanticism (*risōha* 理想派) instead. The debate presented the Japanese public for the first time with the possible roles a modern literature might play. *Bungaku*, the word for literature, which previously stood for the study of classics, now also incorporated independent fiction. This was a result of the imitative reception of European intellectual history in an eclectic Eastern-Western mixture specific to modern Japanese literature. While the translator and scholar of English literature Shōyō published mostly in the literary journal *Waseda bungaku* (as professor of Waseda University), the medical researcher and literary figure Mori Ōgai wrote in the journal *Shigarami Zōshi*, which he himself had founded.2The Japanese authors’ names are in the Japanese order with surname first here, yet in the article’s discussion these canonical authors will be named only with their first names (Ōgai and Shōyō), as it is the Japanese custom for famous writers. The citation sources, in contrast, name their surnames only (Mori and Tsubouchi). The debate is not well known in Western scholarship and occupies a minor place in Japanese academic discussion. Karatani Kōjin’s discussion of this debate in his *Origins of Modern Japanese Literature (1993)* was translated into English, and the philologist Sakai Takeshi (in the 1990s and in 2015) authored an eminent study of it. However, its wider impact remains disputed (Karatani 1993, 145–151).

The debate began with Shōyō’s publication on Shakespeare in the newspaper *Yomiurishinbun* on December 7, 1890, in which he argued that Shakespeare portrayed reality and society objectively, and that this explained his greatness, as literature should document real life. He also evangelized for the ideal that literary works should face the difficult challenge of documenting the reality of human beings and society as authentically and accurately as photography. This was the point at which Ōgai reacted, authoring a number of long articles in which he responded to Shōyō’s Shakespeare-reception by introducing the German literary works of Goethe and others, as well as the aesthetic philosophy of the German thinker Eduard von Hartmann whose metaphysical elaboration on the psychological unconscious fascinated him. In reaction to Ōgai’s criticism of a non-metaphysical poetics, Shōyō then offered a few shorter contributions that would nonetheless defend his understanding of literature as realistic.

During Meiji, local readers became more interested in this paradigm change in Japanese literature, and receptive to works with complex plots, and the public became interested in this paradigm change. Most Meiji authors, including Shōyō, agreed that in the Edo-period literature did not portray characters with a deep psychological profile. As a result, readers in Meiji were no longer attracted to typical Edo fiction like *monogatari* 物語, which were about ghosts and characters without complex psyches. As a contrast, the introduction of the Western literary genre of the novel by Japanese translators, like Shōyō from English and Ōgai from German, re-connoted the term *shōsetsu* from the Chinese classics with the modern understanding of fiction as interesting and with an elaborate plot and story.

This paper explores whether the debate of ‘submerged ideals’ (1891–92) between Ōgai and Shōyō illustrates the birth of modern Japanese literature and criticism out of the imitative reception of Western literature and philosophy only. Shōyō’s advocacy of English Renaissance literature represented by William Shakespeare on the one hand, and Ōgai’s defense of German idealism as exemplified by Eduard von Hartmann’s philosophy on the other, were presented as authorities in the modernization of Japanese literature. This imitative nature of Meiji Japanese intellectuals torn between the antagonistic idealistic, Western or in particular German-inspired, or non-idealistic orientation, after England, are presumed to reveal a certain ‘self-colonial’ essence; to refer to the idea of the forced nature of Japan’s modernity by Komori Yoichi (2001). This study will inquire the degree to which the forced introduction of Japanese modernity was constituted out of an eclectic fusion of a pre-existing non-metaphysical Edo culture as paradigmatic and an imitative acquisition of different Western schools in the Meiji era. The aim is to shed light on the idiom of ‘submerged ideals’ (没理想 論想), understood as metaphysical ideals/*risō* after Romanticism as opposed to its realistic ‘submersion’ in the context of reception into Asia/Japan. The argument for the unique eclecticism fusing Eastern and Western literary traditions in this prominent historical discourse will start with a biographical overview of the two main critics, Shōyō and Ōgai. It will then proceed in two steps, inquiring first into the debate around the reception of the English non- idealistic dramas of Shakespeare after Shōyō, and second around the later discussion of the idealistic German philosophy of Eduard von Hartmann by Ōgai.

In his brief excursion into the *botsurisōronso*, the eminent Japanese literary theorist Karatani claims that ‘Shōyō and Ōgai used the term “ideal” with utterly different meanings’, and that Ōgai had searched for a stratified version of Shōyō’s categorization of ‘a meaning, or theme’ (Karatani 1993, 147). He discusses the evolution of the notion of literature in the Meiji period, from Japanese literary history from antiquity onwards to the absorption of Western poetics in mid-Meiji: ‘For the notion of an evolution, deepening, or progress that took place from antiquity through the middle ages to modernity is a basic schema of Japanese literature and literary history from their time in the founding of the third decade of the Meiji period’ (Karatani 1993, 146).

A brief biographical overview of the debate’s participants provides some context for understanding its multilayered and transcultural nature. Tsubouchi Shōyō, born in Gifu-prefecture, studied English literature at Tokyo Imperial University from 1880 to 1883. With a background in the realist tradition as Japan’s first Shakespeare-translator, he was also an important literary theorist who had authored the first manifesto of the poetics of the novel in Japanese, *Essence of the Novel* (*Shōsetsu shinzui*), 「小説神髄」 (1885–86), in which he argued that literature should give an authentic portrayal of human psychology. Shōyō claimed that realism was the key to the ideal new novel. He was also the author of minor novels like *Tosei shosei katagi* 「当世書生気質」(1885/86) and *Saikun*「細君」 (1891), but stopped writing novels three years later after realizing that he could not write the realistic novel he himself had argued for. A Professor of English literature at Waseda University, he turned to theatre (both Western and kabuki), authoring the modern kabuki-play *Kirihitoha* 「桐一葉」(published in *Waseda bungaku* from 1894 to 95). At the same time, Futabatei Shimei had emerged on the literary scene and published *Ukigumo* 「浮雲」(1887–89) which is considered to be the first modern novel of Japan, written in the aesthetics of naturalism.

Mori Ōgai, originally from Shimane-prefecture, studied medicine at Tokyo Imperial University from 1876 to 1881. More a proponent of the Romantic tradition, he was Japan’s outstanding translator of German literature and a major author of modern Japanese literature, playing a founding role in its poetics. He had been raised as a prodigy, having started with the study of Dutch language, later establishing himself as an expert of both medical science as a state servant and as a literary author taking part in the various philological and social debates of his times. When the *botsurisōronsō* occurred, he had just returned from carrying out medical research in Germany. His most famous novella, ‘Maihime’「舞姫」 (‘The Dancing Girl’), was one outcome of this intercultural experience; it tells the forbidden love story between a Japanese man sent to Berlin by the government and a poor German dancing girl called Elise. The novella has entered literary history due to its poetic accomplishment and formulation of the modern Japanese dilemma of East-West modernity. Ōgai preferred to write in the traditional style of *bungo*, even at a time when the linguistic *genbunit’chi* 言文一致 reform had been established and Japanese language had been modernized. Together with the short stories ‘Fumizukai’ 「文づかひ」 and ‘Utakata no ki’ 「うたかたの紀」(both 1890), ‘Maihime’ forms part of his so-called ‘German trilogy’. Later on, Ōgai authored another semi-autobiographical novel in the genre of the German Bildungsroman, *Vita Sexualis* 「ウィタ・セクスアリス」(1909), as well as historical novels like *Abe ishizoku* 「阿部一族」(1913), *Takase bune* 「高瀬舟」 or *Shibue Chusai* 「渋江抽斎」(1916) and translated important works by Johann Wolfgang Goethe (1749–1832) such as *Die Leiden des jungen Werther* in 1894 and *Faust* in 1913.

## 2 Japanese Shakespeare-reception of Non-metaphysical Early Modern English Literature

The ‘debate on submerged ideals’ began in a *Yomiuri* 読売 newspaper-article by Tsubouchi Shōyō in December 1890 with his praise of Shakespeare. This in turn provoked Ōgai’s critical reaction, ‘Shōyōshi no shohyōgo (逍遥氏の諸評語)’, in September the following year in *Shigarami Zōshi,* the literary journal he had founded. This initiated a one-year-long debate in the two new literary journals of Meiji, Ōgai’s *Shigarami Zōshi* and Shōyō’s *Waseda bungaku*. The eulogizing of Shakespeare as an eminent non-idealistic figure in literary history and the introduction of German idealism illustrate how different Western schools of thought competed with each other following the forced opening-up that put an end to the Sakoku-isolation of the Edo period, according to the pattern of Komori’s conceptualization as ‘self-colonialism’.

Ōgai’s response to Shōyō criticized his peer for the non-metaphysical understanding of literature primarily articulated in his Shakespeare-criticism, and introduced the thought of the German philosopher Eduard von Hartmann (1842–1906) as an alternative orientation for writers like Shōyō or the eminent Meiji author Kōda Rohan (1867–1947). Shōyō responded in ‘Shakespeare kyakuhon hyōchū (シェイクスピア脚本表中)’, in the inaugural issue of *Waseda bungaku* in November 1891, by further extolling Shakespeare’s greatness. The English dramatist’s work, more performed than that of any other playwright in the world, can be subdivided into four periods: the first with his poetry and the tragedy *Romeo and Juliet*; the second with his historical tragedies *King John, Henry IV* and *Richard III;* the third with his so- called four great tragedies *Hamlet, Macbeth, King Lear* and *Othello;* and the fourth by *The Tempest* and *Winter’s Tale.* All of these works were translated from English to Japanese by Shōyō (Tsubouchi 1891). Shōyō added that Shakespeare’s works resembled the Creation of Nature *zōka* (造化), and not divine creation, which means that they were not metaphysical (*keijijōteki* 形而上的).

He also introduced Chinese classic thinkers, claiming that ‘the views of Lao, Zhuang, Yang, Ming, Confucian, Buddhist and other philosophies of the past, present and future are also applicable to nature (creation)’ as ‘Nature (Creation) is a thing that is capable of accepting all of these myriad interpretations’ (Tsubouchi 1891, 2). He further called Shakespeare ‘a natural poet’ who lead ‘his audience to the Theatre of the Globe’, referring to his theatre house in London, and whose poetics, representative of the English Renaissance, ‘were different from those of a secular writer, as they were in the nature of a natural poet’ (Tsubouchi 1891, 3) – and hence in opposition to Christian and idealistic writings. Shōyō also referred to an article by the literary historian Edward Dowden (1843–1913), comparing Goethe and Shakespeare to the ocean, to illustrate the unending nature of their literary genius, hence positioning Japan within a British-German comparative European literary history (Tsubouchi 1891). Shōyō’s literary historical argument closes by acclaiming Shakespeare’s tragedy *Macbeth* (1606):

Ideally, those who are great in Japan should find Japan in the script of Macbeth, those who are great in the universe should find the universe in the script of Macbeth, and those who are ten thousand years old should find eternity in Macbeth. The infinite interest of the poetry of the subliterate ideal lies in its oceanic extent (Tsubouchi 1891, 10).

For Shōyō, Shakespeare’s outstanding non-idealistic play of early modern English literature also has timeless relevance for Japan. As its story of gruesome human psychology in medieval Scotland contains no idealistic dimension, but rather depicts the reality of murderous competition for royal power, the play is also important in the Japanese context, Shōyō argues. He calls the absence of higher values in the play ‘submerged ideals’ as its grandeur was reflected in the awesome natural power of oceans and lakes, both impressive and frightening: ‘But Shakespeare is the greatest poet of all time. He is as boundless as his creation, as broad and deep as his oceans, as deep as his unfathomable lakes, and as universally accessible to popular ideals as any poet before him’ (Tsubouchi 1891, 4).

The comparison of Western literary history with the vastness of the ocean is furthered in an appraisal of other great authors such as Goethe and Walter Scott (1771–1832):

The world is like an azure sea without a shore. It is like a lake without a bottom. It is like a lake whose bottom is unknown; it is deeper and deeper, and its depths cannot be determined at last. Homer, Shakespeare, Goethe, Scott, Eliot, and others are poets of this school, though they are all of different sizes (Mori 1891a, 5).

Possibly, the metaphor of the ocean explains the notion of ‘submerged ideals’, as they might be imagined to be drowned in unending depths, a vastness that needs to be aimed for by Japanese writers of the Meiji era.

Ōgai, in a later contribution, adds the genre of drama to the categorization of the schools to poetry, ‘lyric poetry and world-view poetry’ as affecting the ‘dramatist’, establishing the hierarchy of ‘lyric poetry, epic poetry and drama’ (Mori 1891a, 5). After this categorization of drama and poetry according to the criteria of the ‘submerged ideals-debate’, he returns to the topic of Hartmann and his lack of differentiation between idealism and pragmatism. He is finally focusing on the question of ideals: ‘The power of the revolutionary lies in the small ideas of the heavens and the earth.’ In his criticism of Shōyō’s categorization of poetry, Ōgai also refers to the genuinely Japanese genres ‘from tanka and nagauta at the top to renga, haikai, works of music and joruri at the bottom (except for some pure music), they mostly belong to ideal poetry (lyric school)’ (Mori 1891a, 9). The article closes with reference to Aristotle, whom he had introduced earlier, to illustrate Aristotle’s Greek philosophy as ancient as the Chinese classics, hence establishing a parallel in the segregated literary histories of East and West, in reaching out to the respective ancient origins.

Responding to Shōyō’s promotion of the ‘submerged ideals’ in Shakespeare’s non- metaphysical literature, Ōgai, referring to the German literary history instead of the British, further claims that a truly great poet must nonetheless be idealistic. Paradoxically, Ōgai also ascribes a metaphysical dimension to Shakespeare, while combining this with the idealistic teachings of von Hartmann, also called in Japanese as Dr. Uyu. At the end of the article Ōgai clearly distances himself from the submerged ideals of non-metaphysical conviction as expressed by Shōyō in *Waseda bungaku* to circle around the notion of idealism as distinct from ‘ideas’. At the same time, he distinguishes between ideals and ideas. Strictly speaking, the word *risō* means ‘ideals’, but is used interchangeably with ‘ideas’ throughout the debate.

Shōyō responded a few months later, articulating his atheism as a non-belief in creation and affirms ‘ideals’ as a counter to Ōgai’s call for metaphysics: ‘But I am an obstinate, foolish man, and cannot believe that God exists. (…) I call it the “lost ideal”’ (Tsubouchi 1892c, 3). He continues his anti- metaphysical self-positioning towards ‘ideals’ out of his reception of the English Renaissance within the thought of realism. In the end, he again combines the question of ‘lost ideals’ with poetry to characterize Shakespeare not as an unidealistic writer, but as a poet whose work is compatible with the didacticism of realism:

The same is true when we use the term lost ‘ideals’ in relation to poetry. It means that the writer is lost and invisible. It does not necessarily mean that the writer has no ideals. This is generally the same as when we speak of creation, but there is a little difference. For example, to say that Shakespeare is not ideal does not mean, of course, that he has no ideals, but it does not mean that he has ideals or great ideals that are greater than human beings (Tsubouchi 1892c, 4).

For Shōyō, Shakespeare’s notion of idealism is detached from its common understanding, and he explains the metaphysical interpretation as one out of a possible many. In ‘Ware ni shite nanji ni aru’ (‘What I don’t have but you have’), published in Volume 3 of *Waseda bungaku* from November 1891, he argued that Shakespeare’s work was great because it expressed ‘submerged ideals’ (*botsurisō*), which could be understood as the absence of ideals in the English Renaissance (Tsubouchi 1892a). His primary interest, emerging from his pioneering translations, was to advocate for the cultural importance of Shakespeare to Ōgai, whom, one can assume, in coming from the German intellectual tradition, was not so familiar with the relevance of the English Renaissance. Quite defensively, in reaction to Ōgai’s criticism, Shōyō proposes that his previous comment on Shakespeare was only partially an argument for Shakespeare’s ‘sublime ideal’. Shōyō closes his argument on the ‘submerged ideals’ by advocating for Shakespeare’s non-idealism as one differing from the Christian Romantic literature propagated by Ōgai (Hasegawa 1962), whom he addresses directly at the end of the article.

The debate over idealism or non-idealism in Meiji Japan also took place with the background of Anglo-American colonialism in the Victorian age that Japan almost became a victim of after the forced opening through the Perry-ships in 1854 and the subsequent establishment of the unequal treaties. As a counter-model figured the German Empire that remained widely exempt from colonialism (except for minor territories in South-West Africa) due to its delay in national unification, which provided the model for the Meiji Constitution in 1890, immediately before this debate, and also out of sympathy for the leading German medical and military status without significant territorial expansion. It should also be mentioned that Japan at the time of the debate stood at the turning point of becoming an imperialist power of its own within East Asia, having begun to acquire colonies in Taiwan, and later in Korea and Manchuria.

## 3 Eduard von Hartmann’s Philosophy of German Idealism in the Discourse Around Japanese Modernity

Mori Ōgai introduces to the debate Eduard von Hartmann from the beginning of the sequence as idealist philosopher of aesthetics from Germany; a metaphysical Western thinker who was fully unknown (and untranslated) in Japan at that time. Commonly referred to as Dr. Uyu in Japanese, this minor philosopher had propounded a metaphysics based on the true, the good and the beautiful. His German philosophy and aestheticism, as advocated by Ōgai, figure as an antagonistic model for a modern Japanese literature and non-colonial Western model of modernity as opposed to those of the openly imperialistic English or French. To counter Shōyō’s argument that Shakespeare stood for a non-idealistic literature, Ōgai again referenced the aesthetic philosophy of Hartmann, which in its time had been criticized by the anti- metaphysical philosopher Friedrich Wilhelm von Nietzsche (1844–1900). Hartmann was more famous than Nietzsche for several decades; only when Nietzsche achieved popularity in his later years did Hartmann decide to react to Nietzsche’s accusation of naivety on his part. This did not necessarily refer to aestheticism, but to the idea that there was no beauty in the subjective perception (‘*subjektive-idealistisch*’) of the real world. Hartmann argued that the beauty in arts and music as perceived by the psychological unconscious could be expressed in words.

Furthermore, at the beginning of the debate, Ōgai had distinguished between three schools of the novel in allegiance to Shōyō: the ‘individual’ or ‘unique’ (koyūha 固有派), the ‘eclectic’ (settchūha 折衷派) and the ‘humanist’ (ningenha 人間派) school (Mori 1891a, 8). Referring to authors in the first category, he argues that these hold ‘the theory of heavenly destiny as the ideal’ (Mori 1891a, 3). For those in the second, in which he partially includes the literary luminaries Walter Scott (1771–1832) and Charles Dickens (1812–1870), he claims with some hesitancy that ‘the person is the main object of observation’ (Mori 1891a, 3). Ōgai concludes with the third school, in which he counts Goethe and Shakespeare, and a discussion about cultural and historical changes and literary evolution: ‘The humanist school regards people as the cause and things as the fate, and the cause of this is human nature and the fate of these things is change’ (Mori 1891a, 3). At the same time, he questions such a categorization by asking if a subdivision of the world of literature into schools of fiction really makes sense. His subdivision of novels is also influenced by Eduard von Hartmann’s aesthetic philosophy, which he uses to oppose Shōyō’s poetics: ‘However, the three elements of specificity, eclecticism and humanity are the three schools of thought that Shōyō established. The three categories of ideas – analogy, individual thought, and small world thought – were divided by Hartmann to form a class of beauty.’ The universal classification of novels by Shōyō between ‘the unique school of similar thoughts, the eclectic school of individual thoughts, and the human school of small thoughts about heaven and earth’ (Mori 1891a, 3) inspired Ōgai to insist on the third category as the ‘school of small ideals’. He then writes about the significant philosophical modernist influence by figures like Charles Darwin (1809–1882), whose work was used to develop the theory of Social Darwinism in Victorian Britain as a justification for the expansion of the Empire, in claiming that he transcribes ‘the truth of the world’ that count for all philosophical traditions. Expanding on his reference to Darwin, Ōgai adds: ‘Shōyō says that the school of analogy is like common sense, the school of individual thought is like science, and the school of small ideas is like philosophy.’ Ōgai introduces Hartmann’s aesthetic philosophy that also contains a subdivision of literary fiction: ‘Hartmann’s division of the three categories of beauty into analogy, individual thought and small heavenly and earthly thought is rooted in the fundamentals of aesthetics’ (Mori 1891a, 5).

The philologist Sakai also retraces how Shōyō started the debate with his Shakespeare-criticism in including Japanese literary critics who were inclined to ‘small ideals’ as opposed to Western metaphysics. Sakai inquires into how Ōgai developed the idea(l)s after Eduard von Hartmann’s aestheticism, after Shōyō’s questioning whether these are Platonic, signifying that the idiom of ‘ideal’ (*ris*ō 理想) could be understood as ‘idea’ (*rinen* 理念). Hence, ‘*botsurisō*’ meaning ‘submerged ideals’ stands for the resentment of metaphysical values in modern poetics. Sakai further claims that Ōgai’s reliance on the term also references Georg Wilhelm Friedrich Hegel’s (1770–1831) *Idee* (Sakai 2015, 3). Scott Weinberger, citing the Japanologist Richard Bowring, claims that Ōgai ‘aimed at an objective ideality his criticism expressed through the subjective rhetoric of its language. His theory of art also differed very little from Shōyō’s conjecture that many ideas lay submerged in Shakespeare‘s texts to be activated by the interpretive engagement of the critic’ (Weinberger 2009, 9). He therefore highlights how similar the positions of the two writers were despite their opposition in this debate, that circled around the questions of idealism and Shakespeare as regarding their relevance for the constitution of modern Japanese literature.

Sakai also delves into Shōyō’s classification of literature around the notion of common sense in another categorization: ‘Shōyō says that the school of analogy is like common sense, the school of individual thought is like science, and the school of small ideas is like philosophy’ (Mori 1891a). Ōgai associated Shōyō with Hartmann for an aesthetic philosophical development: ‘However, the three elements of specificity, eclecticism and humanity are the three schools of thought that Shōyō established. The three categories of ideas – analogy, individual thought, and small world thought – were divided by Hartmann to form a class of beauty’ (Mori 1891a, 5). Ōgai continues to explore Hartmann’s aesthetic philosophy, which he had encountered while in Germany and was not translated into Japanese (except for an attempt by Ōgai himself). His recapitulation of Hartmann’s ideas circles around the notion of the individual with an erudite unconscious:

Individuals are high, but not in the same class. Individuals are embodiments, and similes are abstractions. The highest and lowest individual encompasses and forms a segment of the individual below it, just as it encompasses and forms a segment of all the individual things in the world of heaven, earth and thought (Mori 1891a, 6).

In the above, Ōgai deduces from Hartmann a philosophy of the universe that leads to the genesis of individualism as a new Western subjectivity; his discovery of it is the main theme underlying his Romantic semi-autobiographical novella ‘Maihime’ (‘The Dancing Girl’), which was inspired from his four years stay in Germany from 1884 to 1888. Arguing from the position of Romanticism, as it was introduced in Meiji, he assumes the individualistic ideal as the central criterion for modern Japanese literature. He continues with the categorization by associating Shōyō’s poetics with von Hartmann’s: ‘Shoyoko belongs to the two schools of lyricism and world affairs, and Hartmann to the two schools of lyric poetry and epic poetry’. His aspiration to enter a German school of literature and thought in adherence to Goethe, next to Shakespeare, Friedrich Schiller (1759–1805) and Jean Paul (1763–1824) but also Homer, referencing Shōyō, was made explicit at the beginning of the debate (Mori 1891a).

In his short and defensive reaction one month later, in January 1892, Tsubouchi Shōyō writes in ‘Ware ni arazu shite nanji ni ari 我にあらずして汝にあり’ (‘What I don’t have, but you have’) that he dedicated a section of *Jibun Hyōron* (*Review of Contemporary Literature*) to the vitality of Meiji literature. In another contribution to the *botsurisōronsō* from January 15, 1892, in *Shigarami Zōshi* in ‘Uyūsensei ni shasu う鳥先生に謝す (Apologizing to Dr. Uyu)’, Shōyō explicitly opposes Ōgai’s theoretical position based on Hartmann’s: ‘In the *Shigarami- zōshi,* there is a story about a teacher called Uyu-sensei. He opened his exquisite aesthetic eye to see through the theories of Shoyo’s lost ideals, and by using all sides of the argument, he was able to decide on the merits and demerits of the theories.’ (Tsubouchi 1892b, 16). Controversially, he also distances himself rhetorically from the notion that the absence of ideals in a work of literature is a deficit: ‘The ideal of “no-ideal” is a means to absolute despair that does not seek its great ideals’ (Tsubouchi 1892a). This position is further developed in the successive publication about ‘submerged ideals’ on January 30, 1892, ‘Botsurisō no gogi o benzu 没理想の語義を弁ず (Advocating the significance of submerged ideals)’, in which Shōyō insists on a realistic non-metaphysical poetics for a work of fiction to entail an ideal: ‘I mean “idealism lost”, or “idealism not to see”. This can be interpreted as the meaning of “no ideal”, but my intention is the complete absence of ideals, which is naturally different from the meaning of “no ideal”’ (Tsubouchi 1892c, 3–4). Herewith, he distances himself from Ōgai’s embracing of metaphysics and positions himself as a non-idealist, associating himself with the poetics of realism. At the same time, Ōgai expands upon the history of German idealistic philosophy with Arthur Schopenhauer (1788–1860) and Immanuel Kant (1724–1804) to ground his argument in the German Enlightenment towards the end of the debate: ‘All philosophers must know that it is impossible for them not to be biased because of Shōyō. A man like Schopenhauer, who had strong feelings of good and bad, or a man like Kant, who lived in a remote area and had little experience, should not be regarded as a bitter, backward, and perverse person’ (Mori 1892a). Later on, Ōgai affirms his reliance on eminent figures of the German Enlightenment by referring to Gottfried Wilhelm Leibniz (1646–1716), and later Gotthold Ephraim Lessing (1729–1781) for an explanation of his philosophical argument on the monotheistic Christian dualism of good and bad, within which Japan, according to him, must orient itself (see: Mori 1892b). In the same essay, he includes music accompanying the performance of plays by Schiller and Shakespeare, in which he also declares German literature to be more emotional, or more idealistic, when compared to the British: ‘If you compare the music of Schiller with that of Shakespeare, you will see that in Schiller’s music there is more of the writer’s emotion and in Shakespeare there is more of the writer’s viewpoint’ (Mori 1892b, 15).

Furthermore, in ‘Botsurisō no gogi o benzu’ from January 1892, Shōyō adopted a skeptical position towards the Meiji Japanese orientation after great individualist Western writers like Goethe, Turgeniev or Flaubert. He questions whether the divine creation of the earth can be named with idealism as a ‘place where individual people see and feel that it is the heart of creation’ (Tsubouchi 1892c, 3). In referencing Shōyō, Ōgai then compares two main philosophers for this debate, namely Plato and Hartmann, drawing parallels from Greek Antiquity to German idealism: ‘Plato and Hartmann are ideals that have left the shackles of time, and are thought without consciousness. But while Plato used his ideal as an image of this world, and this world as an illusion, Hartmann used his ideal as a non-existent image of this world’ (Mori 1892b, 10). He inquires whether Plato’s philosophy ‘can be taken as an ideal?’ Ōgai thinks that one should pursue an induced understanding of the real world. For him, a Buddhist enlightenment is impossible. Again, he alludes Hartmann’s late work *Philosophie des Schönen (1887)* on the beauty of artistic work. Ōgai had read Hartmann’s *Philosophie des Unbewussten (1869)* while in Germany and interpreted it as a criticism of Darwinism out of the tradition of German idealism. Later, when he returned to Japan, he recognized it as important idealistic philosophy. Hence emphasizing Ōgai’s advocacy of Hartmann’s ideals, it seems that Shōyō cannot really reason against his argument. In the end, Shōyō did not substantially respond to Ōgai’s Hartmann discourse, although one would have expected him to react to the arguments leveled against his ideas. Despite the titles of his contributions to the debate, such as ‘Uyu-sensei ni shasu う鳥先生に謝す’ (‘Apologies to Dr. Uyu’) of January 1892, and ‘Uyu-sensei ni tou う鳥先生に問う’ (‘Asking Dr. Uyu’) in February 1892 (Hasegawa 1962, 50; Tsubouchi 1892b/1892e), it remains a matter of speculation as to whether Shōyō was simply unable to read Hartmann, whom Ōgai had studied in Germany, as it had not been translated from German except for an attempt by Ōgai himself and was not well known in Japan at the time. The arguments of the two thinkers in this debate do not necessarily fully react to each other in a logical and rhetorical structure, which makes it difficult to trace the arguments in the exchange.

## 4 The Context of Edo Japan’s Preservation in the Westernised Aesthetics of Meiji

Shōyō’s eulogy of Shakespeare refers to the comparative theatre history of the seventeenth century. Within the eulogising passage, he evokes Chikamatsu Monzaemon (1653–1725) to make comparisons between Western and Japanese theatre. In praising British and hence Western literary history, he puts forward the different European schools of literature and modernity, English and German (or French), as possible models for Japan’s future path. His anti-metaphysical argument yet ends with this statement on non-idealistic literature in saying that we should rather only praise its lack of ideals. Shōyō also compares Shakespeare’s play *King Lear* (1606) with the work of Edo author Takizawa Bakin/Kyokutei (1767–1848). In addition, he emphasizes how Bashō’s Edo writing was influenced by Murasaki Shikibu from the Heian era: ‘Bashō’s various interpretations of the haiku in The Old Pond are equally reasonable, as are his various misinterpretations of the true meaning of *The Tale of Genji* 「源氏物語」(*Genji Monogatari*)’ (Tsubouchi 1891, 8). Ōgai, in his response, takes issue with this comparison of Shakespeare’s work (*King Lear* in particular) with Chikamatsu and Bakin, relating it to the notion of idealism.

Shōyō, who was a specialist not only of theatre generally, but kabuki specifically, identifies the Edo Japanese kabuki author Chikamatsu as a possible Japanese equivalent of Shakespeare, thus comparing their respective works:

I have been secretly suspicious of this point and have attempted to analyse and dissect Chikamatsu’s works, following the critical methods of his predecessors, such as *Ten no Amijima, Abura Jigoku, Koi Hippo* and *Datezome Tezuna*, all of which are more than capable of accommodating my small ideals. According to my own analysis of the year, the ideals of “Heaven’s Net Island” are between those of *Romeo and Juliet* and their brothers, and the even funnier *Abura Jigoku* almost surpasses the ideals of *The Tempest*, which was also written by an idealist (Tsubouchi 1891, 9).

Shōyō concludes his philological comparison between the English Renaissance and Edo Japan with the insistence that Shakespeare is certainly not inferior to the *jōruri* author Chikamatsu, as his outstanding literary scope reaches back to antiquity:

The number of artists who have been involved in the production of *joruri* is far greater than the number of Shakespearean authors. This is why Chikamatsu’s stories are similar to Shakespeare’s works and are very natural. This being said, it is not to say that Shakespeare’s works are inferior to those of the Joruri authors (Tsubouchi 1892c, 3).

At the same time as he draws comparisons between Edo kabuki and English Renaissance theatre, he insists on Shakespeare’s superiority as a writer over Chikamatsu. His eminence is again illustrated with the Creation of nature or the sea:

Shakespeare never ceases to depict the loftiest ideals in his poetry. But I am still in amazement. But Shakespeare is the greatest poet of all time. He is as boundless as his creation, as broad and deep as his oceans, as deep as his unfathomable lakes, and as universally accessible to popular ideals as any poet before him (Tsubouchi 1891, 10).

Ōgai’s agreement with Shōyō, in reference to Chikamatsu, deplores the fact that Japanese literary history has not brought to life an author as great as Shakespeare or Goethe. He then classifies European authors like Honoré de Balzac, Victor Hugo, Emile Zola and Edward Dowden as part of the humanistic school of literature. Homer is compared with Shōyō’s reception of the Chinese classics, to assert a perceived lack of individuality in Eastern culture: ‘The spirit of the East has never been able to acquire this individualism. This is one example of the theory that there are no individual thoughts in the Orient. Are there really no individual thoughts in our poets? Are there no individual thoughts as in the poems of Goethe and Shakespeare?’ (Mori 1891a, 3). He also addresses the classification of literature in schools, describing two schools of poetry: ‘The first is called the lyric or idealistic school, and the second is called the world-view or creationist school’ (Mori 1891a, 3). This incites him to add a qualitative judgment on the inferior quality of the original school of writings: ‘The reason why they belong to the original school of old writers is because they are mediocre’ (Mori 1891a, 4). New writers belong to the eclectic school because they are too minor in world literary history.

Ōgai’s lament that Japanese literature has not produced a great writer like Shakespeare or Goethe is also contextualized with the humbleness of his own achievements*.* Chikamatsu is however raised as a possible Japanese equivalent to the Western greats because his unidealistic work was of a literary quality of unending reach: ‘When an author has no ideals, he is like a god, a saint, or a perfect man. Chikamatsu, too, is an ideal’ (Mori 1891b, 3). In responding to Shōyō’s comparison of the Edo dramatist Chikamatsu Monzaemon with Shakespeare*,* Ōgai opens up a possible analogy between Japanese cultural heritage and the praised Western tradition: ‘Is Shakespeare the great Chikamatsu Monzaemon?’ (ibid.) Offering abundant references to Western writers like George Eliot (1819–1880), Schiller and even Homer, Ōgai in turn elucidates the complexity of the humanist school for modern Japanese literature. After mentioning another debate he had been involved in, an exchange with the art historian Toyama Masakazu, he goes on to say that Shōyō has developed two schools of poetry in addition to the three schools of fiction. Then, Ōgai references a broad range of canonical European, primarily English, literary history, spanning from Dante Alighieri (1265–1321) to Christopher Marlow (1564–1593), John Milton (1608–1674), Thomas Carlyle (1795–1881), Lord Byron (1788–1824), William Wordsworth (1770–1850) and Robert Browning (1812–1889) as lyric poets, to argue that they match this category (ibid.). Shōyō published three more articles, ‘Kohitsuji ga hakuchūmu 子羊が白昼夢‘, in *Waseda bungaku,* Volume 9 in February 1892, ‘Uyūsensei ni tohu う鳥先生に答ふ’ in Vol. 9/10 and ‘Igi wa chigaeri‘, Volume 10, 1892 in the same month, which together further elucidate a cultural theoretical position and return to the question of ideals among scholars and poets (Tsubouchi 1892d).

In *Shigarami Zōshi*, in turn, Ōgai finally responds with a direct address to Shōyō’s argument on idealism in March 1892, defending the general necessity of metaphysics in literature:

Shoyoko wants to set aside ideals. What is this ideal that you want to set aside? I answer. It is the name given to what individual thinkers, whether they are small thinkers, ordinary people, or even great thinkers in the eyes of the world, have determined to be the heart of creation and the ultimate goal of creation. When such ideals are abandoned, it is safe to say that they are not ideals at all (Mori 1892a, 17).

After this criticism of non-idealistic poetics, Ōgai mentions the Russian writer Ivan Turgenev (1818–1883) as another important Western model for modern Japanese literature and while raising Bakin’s poetry from the Edo period as a pillar for a poetical orientation that might explain Japan’s lack of any author as great as Shakespeare. Reasoning that poetry derives from ‘the unconscious’, he claims that truly valuable literary works must be individualist, like the ones of Shakespeare. The Western orientation of the Meiji literary canon illustrates Japan’s ardent inquiry towards its own path – of an eclectic Asian modernity oriented around the British and the German (or partially French and American) models.

Paradoxically, Shōyō claims Shakespeare to be both idealistic and devoid of idealism (*botsurisō*), suggesting that imitation is inferior to divine creation: ‘We do not call the unconscious creation of the gods “imitation” just because it is accompanied by the “prejudice” of complacency.’ The specificity of the notion ‘to imitate’ for East Asia is also pointed out in saying that the term is derived of the Chinese scholars’ school (Mori 1892c, 3). Ōgai then laments about the notion of the flaneur as a poetic figure regarding *Waseda bungaku*, Shōyō’s forum. In opposition, he retraces the understanding of ‘ideals’ from Plato to German romanticism of the nineteenth century: ‘As I see it, the word “ideal” has undergone many changes from Plato to the present 19th century. If you were to ask me what the ideal is, I would answer that it is the ideal of metaphysics of the 19th century’ (Mori 1892c, 4). He also proposes the Scottish historian Thomas Carlyle (1795–1881) and the American philosopher Ralph Waldo Emerson (1893–1882) as eminent intellectual authorities, ‘priceless’ in a way that Goethe would have appreciated them, again juxtaposing British and German intellectual histories**.** The usage of the concept of ‘ideals’ – the main point of opposition between the two writers – remains contested throughout the debate, as Ōgai associates it with Christian notions of eternity and creation. His ongoing criticism of Shōyō and his ‘emptiness of mind’ then circles around the idea of the ‘tabula rasa’, before finally coming to the teachings of Confucianism.

In his exchange with Shōyō, Ōgai does in fact reason about the use of ‘idealism beyond the Western world’, but prefers to call it the ‘ideal of falling away’ (Mori 1892c, 6). He goes on to consider ‘nothingness’ and the ‘ideal-less ideal’, to insist on the idiom of ideals as something related to Shakespeare’s classic work. The questioning of the terms ‘ideals’ in its Western usage have shifting semantics in reference to Ōgai’s generalizing idiom of ‘an American’ who stands for the Western tradition of thought on idealism in a way that this impacted Japan. The latter figures as a symbol of the Western standard that impacted Japan so much since the forced opening of the country by the US-American Perry ships in 1854:

Who but Shoyoko would dare to name something that has never been called ideal by aesthetics scholars of the past and present? If you call it an ideal, it is based on the words of an American, but you must be aware of the fact that the term ‘ideal’ has been used by aestheticians of the past and present, and that you have no intention of creating a new word for it (Mori 1892c, 7).

Here, the timeless nature of the idiom of ‘ideals’ is sustained as valid for contemporary usage. In ‘Waseda bungaku no botsurironsō’ 「早稲田文学の没理想」(‘The submerged ideals- debate in Waseda literature’) in Volume 27 of *Shigarami zōshi* from December 1892, Ōgai claimed that Shakespeare was great not because he represented submerged ideals, or realistic non-ideological poetics as claimed by Shōyō, but because the thoughts he expressed were truly individualist; a notion Ōgai associates with Hartmann’s philosophy of the unconscious. Subsequently, Shōyō in ‘Botsurisō no gogi wo benzu’ 「没理想の語義を弁ず」 (‘Arguing for the meaning of the submerged ideals-debate’) proposed that the works were still idealistic in their own way (Tsubouchi 1892c). In ‘Waseda bungaku no go botsurisō (The submerged ideals after Waseda literature)’, some months later, Ōgai responded by criticizing Shōyō’s understanding of philosophical ideas as detached from literature (1892b).

The debate circles around the different understandings of the concept of idealism, be it realistically by Shōyō or metaphysically by Ōgai. In the end, as Karatani has pointed out, Ōgai maintains a much stronger position as a successful writer and modernizer. While the debate seems to have rather been won by Ōgai (if there can be said to be a winner), closed as it was with his long contribution from June 1892, to which Shōyō did not further react, the eventual shift of modern Japanese literary history towards realism and naturalism suggests that Shōyō’s argument had a longer-term impact. The exchange of ideas had revolved around how Japanese literature was supposed to be either in the Edo Japanese tradition, with some influence from English realism as advocated by Shōyō, or as metaphysical after the poetics of German idealism in reference to Hartmann as it was expounded by Ōgai. It closes with Ōgai’s criticism of Shōyō’s assumed adherence to the French naturalism of Emile Zola (Hasegawa 1962, 51), whom he also calls an idealist, giving way to the endorsement of the late Meiji emergence of the *shi-shōsetsu* (the Japanese I- novel that equalizes writing and fiction of an author who is part of the *bundan*, the literary establishment). Karatani defends Shōyō and criticizes Ōgai, whom he accuses of having caused the controversy, in their conception of ‘submerged ideals’ for literature since Shakespeare up to the naturalism of Emile Zola, yet also acknowledges that Shōyō was ‘overpowered’ by Ōgai at the end of the debate (Karatani 1993, 148). To this end, Karatani insists on the mirror-function of Shakespeare for what the authors call ‘submerged ideals’, even as they use the notion with ‘utterly different meaning’, and he emphasizes that Ōgai’s inclusion of Eduard von Hartmann is not about German philosophy as such, but merely figures as a point of reference for German idealism in the context of modernizing Japan. The debate’s abundant and rich references to East Asian and Western intellectual and literary history illustrate the dynamic nature and vitality of modern Japanese literature when it was founded out of different poetic traditions in the mid-Meiji period, just as the political construction of eclectic modern Japan was born out of the rapid and selective importation of different Western epistemes in a very short time.

The ‘submerged ideals’ debate took place at the time of the foundation of the Meiji state with the establishment of the Constitution in 1890, and the same year Ōgai’s ‘The Dancing Girl’ was published. Sakai in fact dominates secondary literature on the debate launched by Shōyō’s Shakespeare-criticism, where Shōyō adhered to British empiricism and Ōgai to late German idealism (Karatani 1993, 9). Citing the scholar Sadatani Shigenobu, Sakai also depicts Shōyō’s traditional aestheticism as adhering to *mono no aware* and under Buddhist influence, as opposed to Ōgai’s Christianity-inspired poetics (Karatani, 96). Kamei Shino also attempts the classification of this debate, the first in modern Japanese literature, presenting it as antagonism from the Japan-centered anglicist Shōyō against the German-influenced Ōgai and his defense of metaphysics (Kamei 1998, 31–32).

## 5 Conclusions

Overall, the debate illustrates how different Western poetics were considered to mark the birth of modern Japanese literature, which was influenced by the eclectic absorption of English, German and French modern models at the same time as the consolidation of the Meiji state. At the time of modernization, both Ōgai and Shōyō, despite their young age and certain intellectual immaturity, had established themselves as relevant philological figures in the literary scene. What had started as a disagreement about theatre ended as a general philosophical debate about existential ethical questions confronting the new Japanese state, with the central point of contention being whether modern Japan should follow an English school of thought marked by realism, or the German-inspired path of idealism. Shōyō advocated a realistic understanding of literature that did not represent ideals but portrayed evil characters in a realistic way, such as in Shakespeare’s *Macbeth*. His own novels, like *Toseishoseikatagi,* also strove towards this model. He defended an English realistic model of literature for Japanese society whereby unethical actions were not condemned, but shown realistically. This accords with the history of Japan’s modernization and its turn from being an almost-colonized nation to an imperial power of its own at the time of the *botsurisōronsō* ‘submerged ideals-debate’. Although Shōyō is sometimes presumed to have lost the debate, one could also consider him as a winner as Japanese literature in 20th century took a naturalist turn, with the rise of the I- novel that just portrays an author’s real life.

Ōgai, by contrast, was a romanticist who had just returned from Germany to defend metaphysical ideas. He propagated the moral and ethical poetics of literature of German Idealism, and as represented by Goethe, Schiller, Leibniz and Lessing. While Shōyō had no explicit philosophical training, Ōgai endorsed the aesthetic and idealistic philosophy of Eduard von Hartmann, who also influenced Sigmund Freud and Carl Gustav Jung. The exchange of arguments of these two major writers around notions of German idealism and English realism illustrates how much early modern Japanese literature was formed by its imitative orientation towards different Western poetic authorities. This process was made possible out of the mass translation of the works of European thinkers into Japanese, and suggests how arduously the intellectuals of those times studied Western history of thought and literature. This is representative of Meiji’s acquisition of an eclectic modernity imported from Europe (and America), in which the ‘submerged ideals-debate’ provided a literary-historical precedent in the constitution of an independent Asian modern nation that could then compete with the West.
